# INSIDIA 2.0 High-Throughput Analysis of 3D Cancer Models: Multiparametric Quantification of Graphene Quantum Dots Photothermal Therapy for Glioblastoma and Pancreatic Cancer

**DOI:** 10.3390/ijms23063217

**Published:** 2022-03-16

**Authors:** Giordano Perini, Enrico Rosa, Ginevra Friggeri, Lorena Di Pietro, Marta Barba, Ornella Parolini, Gabriele Ciasca, Chiara Moriconi, Massimiliano Papi, Marco De Spirito, Valentina Palmieri

**Affiliations:** 1 Dipartimento di Neuroscienze, Università Cattolica del Sacro Cuore, Largo Francesco Vito 1, 00168 Rome, Italy; giordano.perini@unicatt.it (G.P.); enrico.rosa@unicatt.it (E.R.); ginevra.friggeri01@icatt.it (G.F.); gabriele.ciasca@unicatt.it (G.C.); marco.despirito@unicatt.it (M.D.S.); 2 Fondazione Policlinico Universitario A. Gemelli IRCSS, 00168 Rome, Italy; lorena.dipietro@unicatt.it (L.D.P.); marta.barba@unicatt.it (M.B.); ornella.parolini@unicatt.it (O.P.); 3 Dipartimento di Scienze della Vita e Sanità Pubblica, Università Cattolica del Sacro Cuore, Largo Francesco Vito 1, 00168 Rome, Italy; 4 Theolytics, The Sherard Building, Edmund Halley Road, Oxford Science Park, Oxford OX4 4DQ, UK; chiara.mori@gmail.com or; 5 Istituto dei Sistemi Complessi, CNR, Via dei Taurini 19, 00185 Rome, Italy

**Keywords:** quantum dots, graphene, photothermal therapy, cancer spheroids, image analysis

## Abstract

Cancer spheroids are in vitro 3D models that became crucial in nanomaterials science thanks to the possibility of performing high throughput screening of nanoparticles and combined nanoparticle-drug therapies on in vitro models. However, most of the current spheroid analysis methods involve manual steps. This is a time-consuming process and is extremely liable to the variability of individual operators. For this reason, rapid, user-friendly, ready-to-use, high-throughput image analysis software is necessary. In this work, we report the INSIDIA 2.0 macro, which offers researchers high-throughput and high content quantitative analysis of in vitro 3D cancer cell spheroids and allows advanced parametrization of the expanding and invading cancer cellular mass. INSIDIA has been implemented to provide in-depth morphologic analysis and has been used for the analysis of the effect of graphene quantum dots photothermal therapy on glioblastoma (U87) and pancreatic cancer (PANC-1) spheroids. Thanks to INSIDIA 2.0 analysis, two types of effects have been observed: In U87 spheroids, death is accompanied by a decrease in area of the entire spheroid, with a decrease in entropy due to the generation of a high uniform density spheroid core. On the other hand, PANC-1 spheroids’ death caused by nanoparticle photothermal disruption is accompanied with an overall increase in area and entropy due to the progressive loss of integrity and increase in variability of spheroid texture. We have summarized these effects in a quantitative parameter of spheroid disruption demonstrating that INSIDIA 2.0 multiparametric analysis can be used to quantify cell death in a non-invasive, fast, and high-throughput fashion.

## 1. Introduction

The side effects of chemotherapeutic drugs prompted the study of drug delivery systems that more accurately target tumors rather than healthy cells. These systems are often nanoparticle-based therapies that have also been shown to play a role in overcoming cancer-related drug resistance [1].

In this field, the standard model for the in vitro study of cancer cell invasion and response to therapy has been historically the two-dimensional (2D) monolayer cell culture. Although 2D models have provided relevant contributions to tumor investigation, they now have been replaced by three-dimensional (3D) models such as spheroids and organoids [2,3].

Indeed, it has been demonstrated that 2D cultures express distinct markers due to the absence of a proper physiological microenvironment that faithfully reproduces the earliest stages of metastasis [4]. Moreover, interactions among cells and between cells and extracellular matrix are altered in 2D conditions, thus limiting the reliability of these models for therapeutic effects of drug delivery systems [3]. Therefore, the use of effective 3D in vitro models capable of recapitulating in vivo features has started to spread in research laboratories, allowing the analysis of microenvironment, aggressiveness, and tumor progression using specific therapeutic strategies [5,6]. 

The increasing number of applications of 3D spheroids resulted in the possibility to perform high-throughput screening, including large-scale image analysis [6]. Indeed, multiplate readers allow for the acquisition of images from spheroids in multi-wells, increasing the amount of data to analyze. Therefore, there is a need for rapid, user-friendly, ready-to-use, high-throughput image analysis software able to reduce the costs associated with specific technologically advanced systems.

Most of current analysis methods involve manually drawing the width and length of the imaged 3D spheroids, which is a time-consuming process that is extremely liable to the variability of individual operators [7]. Some software tools can perform spheroid separation from the background, i.e., the segmentation of spheroid. The examples available in the literature are SpheroidJ, SpheroidSizer, TASI, and AnaSP [8,9,10,11,12]. However, many tools are focused on segmentation, neglecting multiparametric analysis. Other image analysis tools are licensed for specific microscope platforms [13], lacking customizability by the end-user for full-spectrum analysis [14]. This is extremely important for high throughput testing of new nanomaterials such as nanoparticles, liposomes, and quantum dots, which are recently widely used in cancer research [15,16].

Graphene quantum dots (GQDs) are small semiconducting nanoparticles that have shown high biocompatibility and low cytotoxicity both in vitro and in vivo [15]. Our previous findings strongly indicate that positively charged carboxylated GQDs can act as synergistic enhancers of chemotherapeutic agents, significantly improving the efficacy of drugs and allowing, at the same, time a reduction in drug doses [17]. Furthermore, GQDs can efficiently absorb near infrared (NIR) light and convert it into heat, exerting a strong photothermal killing effect in photothermal therapy (PTT) [18].

In this work, we report the analysis of the effects of PTT mediated by GQDs on two cancer models, i.e., glioblastoma and pancreatic cancer spheroids, obtained with a new version of INSIDIA (Invasion SpheroID ImageJ Analysis) macro [19].

INSIDIA macro has the advantage, when compared to other tools, to allow high end-user customization and the rapid quantitative analysis of entire spheroid datasets [19]. By using INSIDIA, it is possible to isolate the spheroid mass from image background both for bright-field grey and fluorescent images and to distinguish its core and invasive edge, thus providing quantitative information of tumor invasiveness and growth. This analysis is performed in a non-disturbing manner compared to standard biochemical assays, allowing also the differentiation of core and edges features that are merged in disruptive cell analysis.

We have created INSIDIA 2.0 to implement the most recent relevant features described in the literature such as novel structural and functional parameters to evaluate cancer growth, progression, and response to nanoparticle therapy [20]. Here, we demonstrate that INSIDIA 2.0 can retrieve parameters from growing spheroids and also can discriminate several processes induced by the uptake of GDQs including spheroids’ death by collapse and/or spheroid disaggregation. We also propose a new parameter that is intended for evaluating spheroid viability from morphological and grayscale features and demonstrate the high versatility of INSIDIA 2.0 in nanoparticle research for non-invasive analysis of cancer models.

## 2. Material and Methods

### 2.1. Cell Culture

U87MG human glioblastoma cells were purchased from the American Type Culture Collection (ATTC, Manassas, VA, USA). Cells were cultured in Dulbecco’s modified Eagle’s medium (DMEM) (Sigma-Aldrich, St. Louis, MO, USA) supplemented with 10% fetal bovine serum (FBS, Euroclone, Milan, Italy), 2% penicillin-streptomycin (Sigma-Aldrich), and 2% L-glutamine (Sigma-Aldrich). PANC-1 human pancreatic cancer cell lines (ATCC^®^ CRL1469™) were cultured in DMEM with sodium pyruvate (Aurogene, Rome, Italy) supplemented with 10% FBS (ATCC), 1% L-glutamine (Euroclone), and 1% antibiotics (Aurogene). Cells were cultivated in T75 flasks and kept at 37 °C, 5% CO_2_.

### 2.2. Characterization of Graphene Quantum Dots

Carboxylated GQDs in double-distilled water (ddH_2_O) solution with a concentration of 1 mg/mL have been purchased from ACS Material (Pasadena, CA, USA). Optical and spectroscopic characterizations of GQDs were carried out as described elsewhere [15,17,21]. Fluorescence intensity spectra were obtained by using a Cytation 3 Cell Imaging Multi-Mode Reader (Biotek, Terrebonne, QC, Canada), using excitation wavelengths from 260 to 600 nm and acquiring emission from 300 to 700 nm. Fluorescence intensity spectra were normalized to the maximum emission. Then, 100 μL of samples with a concentration of 10 μg/mL was deposited on sterile mica slides and air-dried overnight for atomic force microscopy imaging (AFM) with a NanoWizard II (JPK Instruments AG, Berlin, Germany) [22]. Images were acquired using silicon cantilevers with conical silicon tips (CSC36 Mikro-Masch, Tallinn, Estonia) characterized by an end radius of about 10 nm, a half conical angle of 20°, and a spring constant of 0.6 N/m. Scan areas of 3 × 3 μm were imaged. Dynamic light scattering was performed with Zetasizer Nano ZS (Malvern, Herrenberg, Germany), equipped with a 633-nm He−Ne laser and operating at a scattering angle of 173°. UV-transparent cuvettes (Malvern, Herrenberg, Germany) were used for experiments with a sample volume of 500 μL and a concentration of 100 μg/mL. The measurements were performed at a fixed position (4.65 mm) with an automatic attenuator. The hydrodynamic radius (Z-average size) was obtained by using the Stokes−Einstein equation. Data analysis was performed by Malvern Zetasizer software. Chemical analysis of the nanoparticles was performed using attenuated total reflectance-Fourier transform infrared spectroscopy (ATR-FTIR) by Spectrum One spectrometer (Perkin Elmer, Waltham, MA, USA). The nanomaterial was directly drop casted upon the ATR crystal and the spectra were recorded in the wavenumber range of 4000–550 cm^−1^ [23]. To perform photothermal therapy, spheroids were transferred at every timepoint under an 808 nm laser (Laser Ever). The spot of the laser had a diameter of 0.8 cm. The irradiation of cells was performed for 5 min at a power density of 6 W/cm^2^. Thermal increase was monitored over time by focusing a thermal camera (Optris) on the irradiated spheroids. Spheroids were then incubated at 37 °C with 5% CO_2_ for further analysis.

### 2.3. Spheroid Preparation and Viability Tests

For spheroid preparation, U87MG or PANC-1 cells were seeded on 96-well, round bottom, ultra-low attachment plates (Corning, Corning, NY, USA) at a density of 0.25 × 10^5^ cells/Ml [16]. The plate was centrifuged at 300× *g* for 3 min to ensure the confluence of cells to the center of the wells. The so-formed single spheroids were incubated at 37 °C with 5% CO_2_.

Spheroids in triplicate cultured on 96-well, round bottom, ultra-low attachment plates were treated for 2 weeks with GQDs (200 μg/mL) with or without chemotherapy administration (doxorubicin 1 μM for U87 and 5-Fluorouracil 100 μM for PANC-1). Culture medium was changed with fresh medium containing the same concentration of nanoparticles and chemotherapeuticals every three days. Transmittance images of spheroids were collected using Cytation 3 (BIOTEK) and then analyzed with INSIDIA 2.0. Cell viability was measured after 7 and 14 days by the CellTiter-Glo^®^ Luminescent Cell Viability Assay (Promega, Madison, WI, USA). Briefly, an amount of CellTiter-Glo^®^ reagent equal to the volume of culture medium was added to each well. The plate was orbitally shaken for 5 min; then, it was incubated in the dark at room temperature for 20 min to ensure complete cell lysis. Luminescence was then recorded with Cytation3 Cell Imaging Multi-Mode Reader. Results were normalized by untreated (control) spheroids at day 0. Statistical analysis was carried out by using one-way ANOVA and Turkey post-hoc test. Data were considered significant when *p* < 0.05.

### 2.4. Image Analysis with INSIDIA 2.0

Image analysis has been performed with INSIDIA 2.0 the updated version of INSIDIA [19].

**Figure 1:**
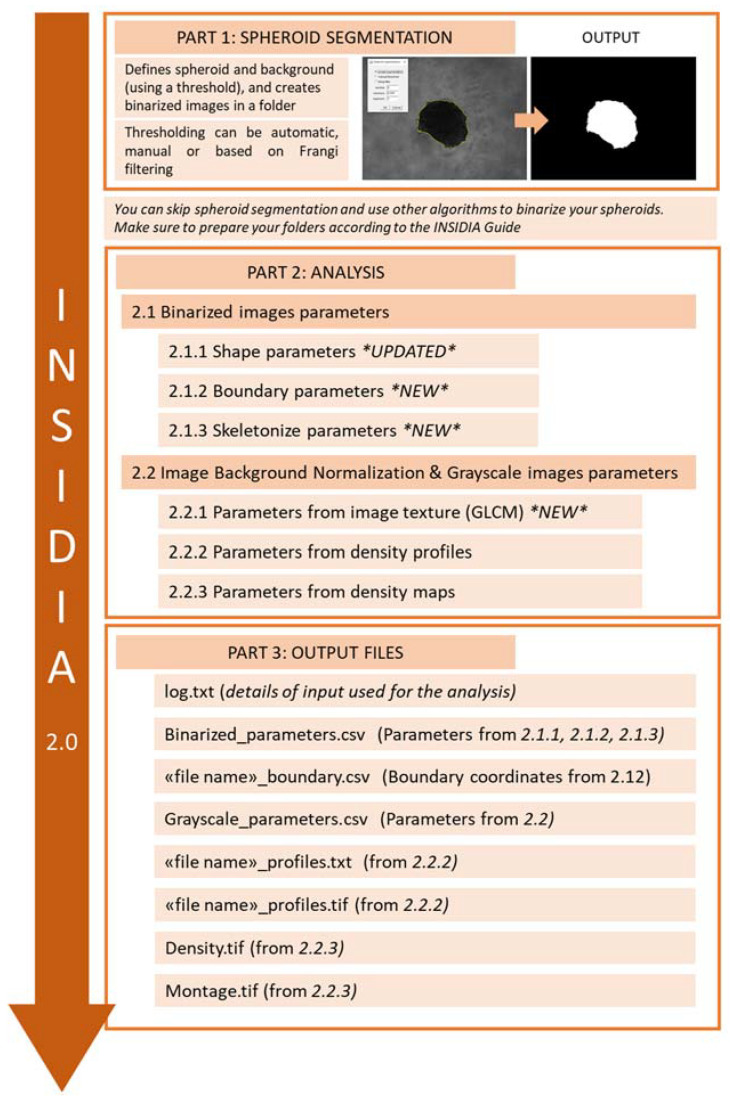
INSIDIA 2.0 workflow comprises spheroid segmentation (optional), analysis of binarized and grayscale images, and generation of output files.

In Figure 1, we show the analysis workflow of INSIDIA 2.0, while in the supplementary files, the INSIDIA 2.0 macro guide is reported. Macro files and test files are available at the following website: https://www.isc.cnr.it/staff-members/valentina-palmieri/ (accessed on 17 February 2022).

Initially, if requested by the user, INSIDIA 2.0 performs Spheroid segmentation (SpS) on grayscale images of spheroids. The aims of SpS are (i) the identification of the spheroid in each image and (ii) the separation from the background by thresholding. The outputs of SpS are binary images containing the spheroid (white pixels) on a black background. Binarized images are saved in a results folder, created during code execution, in each experimental folder. If the user has already provided segmented images (together with original grayscale images), the SpS execution part can be skipped.

The binarized images are used as inputs to obtain parameters from binarized images and, specifically, shape parameters, boundary parameters, and skeletonize parameters (the complete parameters list is available in the Supplementary Files in the Macro guide). After the analysis of binarized spheroid, INSIDIA 2.0 proceeds with the analysis of normalized grayscale images and retrieves data from images grayscale texture.

Finally, concentric circles are created from spheroid centroid towards spheroid edges to perform Density Profile analysis and define the Core Threshold, which distinguishes between the spheroid core and edges [19]. Once CT is calculated, the image is colored accordingly, and the Density Map of the spheroid is created [19].

Spheroid disruption has been quantified using the following equation:

$$\mathrm{Spheroid}\;\mathrm{Disruption}=\frac{Area_{t}-Area_{0}}{\left(Percore_{t}-Percore_{0}\right)\times \alpha }$$

where Area is the total spheroid area calculated from binarized spheroids, Percore is the percentage of core area over total area calculated from density maps, and *α* is the difference of entropy ($Entropy_{t}-Entropy_{0})$ calculated using GLCM.

## 3. Results and Discussion

### 3.1. Characterization of GQDs

GQDs are considered promising nanomaterials for the photothermal therapy of cancer due to their biocompatibility, capability of crossing body barriers, and rapid excretion due to their small size [24,25].

**Figure 2:**
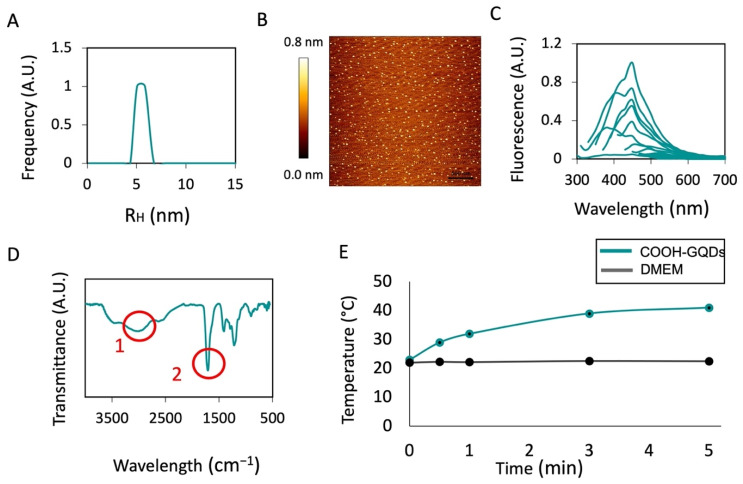
Characterization of GQDs. The hydrodynamic radius of the nanoparticles by DLS (**A**). AFM imaging of GQDs (**B**) with a scalebar of 500 nm. Fluorescence emission spectra of carboxylated GQDs from 300 to 700 nm after excitation wavelengths ranging from 260 to 600 nm (**C**). Data are normalized by maximum excitation. FTIR spectroscopy of GQDs indicating the presence of O-H (red circle 1) and C=O stretching (red circle 2) (**D**). Photothermal conversion of GQDs during a time of 5 min, with an 808 nm laser excitation at a power density of 6 W/cm^2^. DMEM medium has been used for comparison (**E**).

The characterization of carboxylated GQDs has been performed by spectroscopic and microscopic techniques. Dynamic light scattering (DLS) has been carried out on samples at a concentration of 100 μg/mL. DLS analysis shows a GQDs hydrodynamic radius smaller than 10 nm (Figure 2A). Atomic force microscopy (AFM) imaging, performed on samples on mica at a concentration of 10 μg/mL (Figure 2B), further confirmed size distributions obtained from DLS. The maximum measured height by AFM topology was around 0.8 nm, as expected from the literature [26]. Fluorescence emission spectra have been also acquired by exciting GQDs at wavelengths ranging from 260 to 600 nm (step size 20 nm) and recording emission from 300 to 700 nm (step size 5 nm). The maximum fluorescence peak of the nanoparticles was found by exciting at 320 nm and reading emission at 450 nm, in the blue range (Figure 2C). Fourier transform infrared spectroscopy (FTIR) (Figure 2D) depicted two relevant transmittance peaks: one between 3000 and 2800 cm^−1^, indicating the presence of O-H stretches, and another one, more evident, at 1750 cm^−1^, representing the typical C=O stretch of carboxy groups [21]. Taken together, spectroscopic and microscopic characterizations highlight the homogeneous size distribution of the GQDs, along with their specific surface chemical functionalization. Graphene-based materials have widely shown their capability of absorption and conversion of infrared light into heat. NIR absorption band from 650 nm to 950 nm has been observed by Wang et al., which is related to extensive delocalized electrons in GQDs [27]. Importantly, positively charged GQDs were successfully employed both in vitro and in vivo to exert a combination of PTT and real-time imaging [28]. To perform PTT, we first tested the photothermal conversion of carboxylated GQDs (Figure 2E) using an 808 nm laser with a power density of 6 W/cm^2^. We measured irradiation-dependent temperature increases in the solution containing positively charged GQDs diluted in culture medium (DMEM). GQDs reached a temperature of 42 °C in 5 min, while no increase in temperature was measured in the control (culture medium alone), confirming the strong photothermal conversion of GQDs (Figure 2E).

### 3.2. INSIDIA 2.0 Analysis of Treatments on U87 Glioblastoma Spheroids

Analysis of U87 spheroids has been performed with INSIDIA 2.0, which has been improved for the analysis of segmented spheroid images and the analysis of grayscale texture.

**Figure 3:**
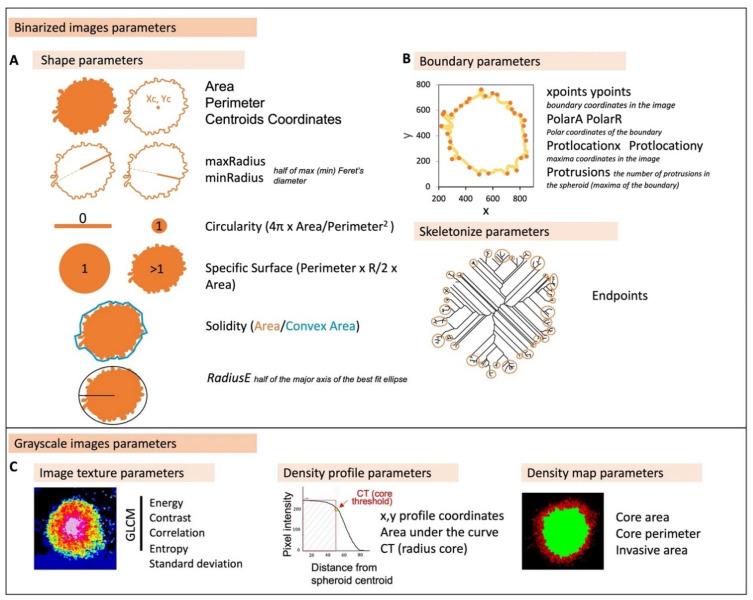
Overview of parameters automatically extracted by INSIDIA 2.0. (**A**) Binarized images parameters include shape parameters as well as (**B**) boundary derived parameters and skeletonized image number of endpoints. In (**C**), parameters from grayscale images are shown: image texture parameters, a new feature on INSIDIA 2.0, and density profile and map parameters.

In addition to code optimization for a high number of files, INSIDIA 2.0 has new features as summarized in Figure 1 in the Materials and Methods section. Parameters obtained from INSIDIA 2.0 can be categorized in binarized and grayscale image parameters (Figure 3A–C).

Binarized image parameters include morphological features and the extraction of spheroid boundary and the quantification of maxima on the boundary profile to measure the number of protrusions as well as the quantification of endpoints on skeletonized spheroids (Figure 3A,B). This boundary extraction is of utmost importance for analyzing epithelial to mesenchymal transition, as reported in recent papers [14] and improves the performance of the original INSIDIA macro.

On the other hand, grayscale features of spheroids have been analyzed to retrieve image texture parameters using gray level cooccurrence matrix (GLCM) [29] and measuring the standard deviation of the gray values, as well as density profiles and density maps, as described previously [19].

U87 spheroids have been treated with doxorubicin (Dox) with or without GQDs administration/PTT therapy.

Although Dox is an effective anti-cancer drug recommended for a variety of malignant cancers, it lacks penetration across the blood–brain barrier; therefore, it is not used for glioblastoma treatment. However, several papers demonstrated the possibility to combine doxorubicin with nanoparticles such as iron nanoparticles to improve its biodistribution after administration [30,31]. In our previous work, we have shown how GQDs have good biocompatibility toward neurons and can improve Dox intracellular administration in 2D cell cultures of U87 [15]. Here, we exploit the light absorptive properties of GQDs and evaluate the effect of a combination of Dox and PTT therapy.

**Figure 4:**
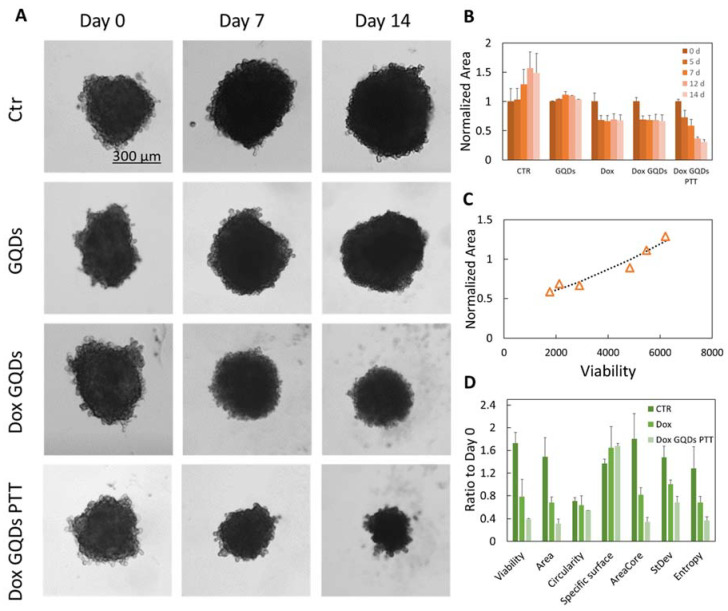
(**A**) Representative images at days 0, 7 and 14 of U87 control (Ctr) spheroids and treated with GQDs, Dox GQDs, or Dox GQDs PTT. (**B**) Effect of treatments on spheroids area, obtained from binarized images and normalized to Day 0. (**C**) Correlation between normalized area and viability after 7 days of treatment and non-linear fit of data with equation ax^3/2^ + b and parameters a = 1.57 × 10^−6^ ± 1.8 × 10^−7^, b = 0.47 ± 0.06. (**D**) Comparison of different parameters obtained with INSIDIA 2.0 after 14 days of treatment.

In Figure 4A, representative images of U87 control spheroids and after GQDs, Dox-GQDs, or Dox-GQDs PTT treatments are shown. The concentration of GQDs was set to 200 μg/mL according to previous data on biocompatibility, while Dox has been administered at a concentration of 1 uM as previously [18]. The viability of spheroids has been assessed after 7 and 14 days of treatment to test therapeutic effects after long-time exposure (Figure S1). Viability data show a significant effect of the combined Dox and GQDs therapy with or without PTT. Data of total spheroid area at several timepoints obtained with INSIDIA 2.0 spheroid segmentation are reported in Figure 4B. After 14 days, the Ctr samples increase their area up to 150%, while treatment with 200 μg/mL GQDs impedes growth without a significant reduction in spheroid size. These data agree with the reduction in cell viability (Figure S1).

The treatment with Dox immediately blocks spheroid growth, and after 5 days, the size of the spheroids is reduced by 30%. This result is similar for Dox GQDs. However, the administration of PTT and DOX reduces spheroid volume by 70% of the initial volume after 14 days. Data are well correlated with viability tests with an R value of 0.929 as shown in Figure 4C.

A direct comparison of viability at 14 days with selected INSIDIA 2.0 parameters in Figure 4D shows the power of multiparametric analysis. Precisely, the decrease in circularity, accompanied by the increase in specific surface, shows how treatments cause a progressive loss of spherical shape. In this case, we can attribute it to the brittleness of spheroid border and a loss of integrity. Concerning parameters obtained from grayscale images, we can observe a reduction in the core area, which follows a global reduction in the total area of spheroids that occurs with treatments.

Parallelly, the standard deviations of the gray levels of pixels and the entropy calculated with GLCM are reduced by treatments. This occurs because the spheroids’ homogeneity increases with death, as visible in Figure 4A images, and further discussed in the following paragraph.

### 3.3. Analysis of Pancreatic Spheroids: The Texture of Spheroids

To demonstrate the efficacy of GQDs-based PTT in other cancer models, we have tested therapy for pancreatic cancer using a combination of GQDs, 5-fluorouracil (5FU) and PTT. 5FU is used for combined treatment protocols of pancreatic cancer [32].

**Figure 5:**
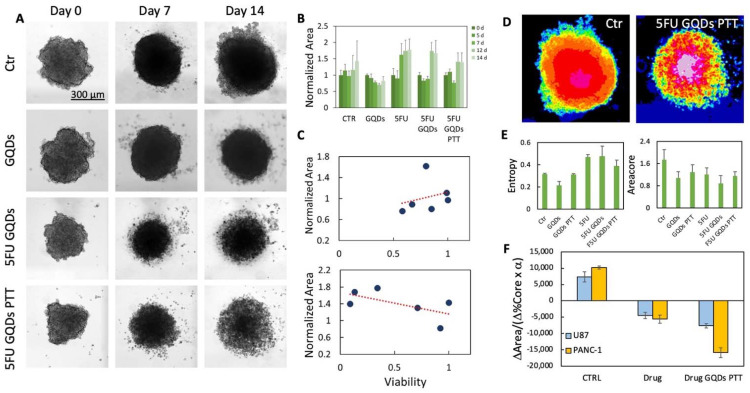
(**A**) Representative images at day 0, 7, and 14 of PANC-1 spheroids treated with GQDs, 5FU GQDs or 5FU GQDs PTT. (**B**) Effect of treatments on spheroids area obtained from binarized images and normalized to Day 0. (**C**) Correlation between normalized area and viability after 7 or 14 days of treatment. (**D**) Representative images of Ctr or 5FU GQD PTT-treated spheroids with 16 colors in the table to highlight differences in grayscale texture. (**E**) The area of the core obtained from spheroids profiles and entropy of spheroids after 24 days of different treatments. (**F**) Parameter quantifying spheroid disruption after 14 days of treatment.

Pancreatic spheroids have been grown for 14 days in the presence of GQDs or GQDs and 5FU with or without PTT therapy. In Figure 5A, the representative images of PANC-1 spheroids are reported at 0, 7, and 14 days after different treatments compared to Ctr samples. The viability of spheroid cells after 7 and 14 days of treatments is reported normalized to control (untreated) spheroids at day 0 (Figure S2).

As expected from the literature, the treatment with 5FU has a good efficacy with a reduction in viability of 20% and 70% after 7 or 14 days, respectively. The use of 5FU in combination with GQDs or GQDs PTT enhances the effect of the drug. Interestingly the treatment with 5FU shows a complete loss of spheroids integrity after 14 days of treatment.

In this case, the parameters obtained from binarized images such as the area of the spheroid (Figure 5B) indicate an opposite effect compared to viability, as if the spheroid is continuing its growth. Consequently, the correlation between viability and area is completely lacking both at 7 and 14 days (Figure 5C).

This occurs because the spheroid loses its compactness with an apparent increase in total area. As a confirmation of this, considering parameters related to grayscale features such as entropy, an overall increase in entropy can be observed in 5FU-treated spheroids after 14 days (Figure 5D,E). At the same time, the core area, which is the intense dark area in the center of the spheroid, is reduced (Figure 5F).

Overall, two types of spheroid death have been described, as summarized in Figure S3. A growing spheroid roughly maintains the core/edges area proportion and increases its volume (Figure S3A). In U87 spheroids, death is accompanied by a decrease in the area of the entire spheroid, with a decrease in entropy due to the high uniform density of the spheroid core (Figure S3B).

For PANC-1 spheroids, death is accompanied by an overall increase in area and entropy due to the progressive loss of integrity and high variability of grayscale values (Figure S3C).

Both types of death are indicators of efficacy of the nanoparticle-based therapy; however, standard morphological analysis might not provide an accurate representation of these phenomena. Consequently, multi-parametric analysis is of fundamental importance to describe spheroids behavior. Precisely, we can hypothesize that an overall stability (or small variation) of entropy indicates a self-consistent spheroid structure. On the other hand, an opposite trend of area growth compared to core percentage evidence a death process, which is either necrotic or disruptive.

We have converged the observed trends in a spheroid disruption parameter in which the variation of core percentage is normalized over the total area variation weighted for entropy (*α*), as shown in Figure 5F. When this parameter is below zero, i.e., there is an opposite trend of total area and core percentage, a death process occurs. This analysis shows how the distinction between core and invasive edges is of fundamental importance and how standard assays could have caused a loss of information given the disruption of spheroid mass during the labeling protocol.

## 4. Conclusions

The ability to relate spheroid morphology and texture to cytotoxic responses is invaluable in screening rationally designed combinations of oncology treatments. Here, we demonstrate that INSIDIA 2.0 can distinguish several effects mediated by cytotoxic drugs, nanoparticles (GQDs) and photothermal treatments (NIR laser).

INSIDIA 2.0 represents a unique open-source tool capable of high-throughput multi-parametric analysis, is easily customizable by the end-user, and is compatible with other segmentation software and all imaging platforms. Spheroid screening with INSIDIA 2.0 will allow the removal of non-efficacious candidate nanoparticles earlier in the pipeline and discriminates subtle changes in spheroids viability without invasive testing with an overall minimization of costs and experimental time.

The spheroid disruption parameter can highlight several types of spheroids’ loss of viability, and future experiments will highlight the significance of different types of spheroid death with spheroids grown into the matrix. INSIDIA 2.0 is fully compatible with specific segmentation software since users can directly provide segmented spheroids. INSIDIA 2.0 shows high versatility even in the field of materials sciences, as well as in precision nanomedicine.

## Data Availability

Macro files and test files are available at the following website: https://www.isc.cnr.it/staff-members/valentina-palmieri/ (accessed on 17 February 2022).

## Supplementary Materials

The following supporting information can be downloaded at: https://www.mdpi.com/article/10.3390/ijms23063217/s1.
