# Supplementary material for: INSIDIA 2.0 High-Throughput Analysis of 3D Cancer Models: Multiparametric Quantification of Graphene Quantum Dots Photothermal Therapy for Glioblastoma and Pancreatic Cancer
Source: Int J Mol Sci. 2022 Mar 16;23(6):3217. doi: 10.3390/ijms23063217 (PMC8948775; doi:10.3390/ijms23063217)
Supplement: Supplementary file 1 [file ijms-23-03217-s001.zip › INSIDIA 2.0 Macro Guide.pdf]

Guide for INSIDIA (INvasion SpheroID ImageJ Analysis)

Version 2.0

Contents

1. Introduction 2

1.1 INSIDIA 2.0 workflow 3

1.2 Input images and folders organization 6

2. INSIDIA 2.0 QUICK guide 7

3. INSIDIA output and parameters 12

4. INSIDIA Code 15

4.1 Functions (lines 1-39) 15

4.2 ImageJ settings & Errors (lines 40-51) 15

4.3 Menu & Folders (lines 52-101) 15

4.4 INSIDIA PART 1: Spheroid Segmentation (lines 102-239) 15

4.6 INSIDIA PART 2: Analysis 16

4.6.1 Parameters (240-278) 16

4.6.2 GLCM Menu (279-286) 16

4.6.3 Shape Parameters from binarized images (287-363) 16

4.6.4 Boundary Parameters from binarized images (364-413) 17

4.6.6 Skeletonize Parameters from binarized images (414-422) 17

4.6.7 Image Normalization and background removal (423-453) 17

4.6.7 GLCM texture analysis (454-462) 18

4.6.8 Saving of normalized images (463-476) 18

4.6.9 Pixel Conversion Menu (476-491) 18

4.6.10 Density Profile (492-540 and 561-607) 18

4.6.11 CT Calculation (541-560) 18

4.6.12 Density Map & Montage (608-704) 19

4.7 INSIDIA PART 3: Output 19

Output, Save & exit (705-752) 19

1. Introduction

This is the User Guide for ImageJ (FIJI) Macro INSIDIA 2.0. Before running INSIDIA, please make sure that you have the latest version of FIJI installed on your PC.

INSIDIA requires the following plugins:

- Concentric Circle (https://imagej.nih.gov/ij/plugins/concentric-circles.html),
- Frangi Vesselness (http://imagej.net/Frangi)
- GLCM (https://imagej.nih.gov/ij/plugins/texture.html).

To run INSIDIA, download the macro code from the website (https://www.isc.cnr.it/staff-members/valentina-palmieri/). Open the script in FIJI (to open the script window go to the menu *File> New> Script*) and select ImageJ macro language in the menu *Language* (Figure 1). To start the code click run.

!Warning! If you click Cancel, in any menu of INSIDIA 2.0, the code will stop. Do not click in other windows while the macro is running or the code might stop

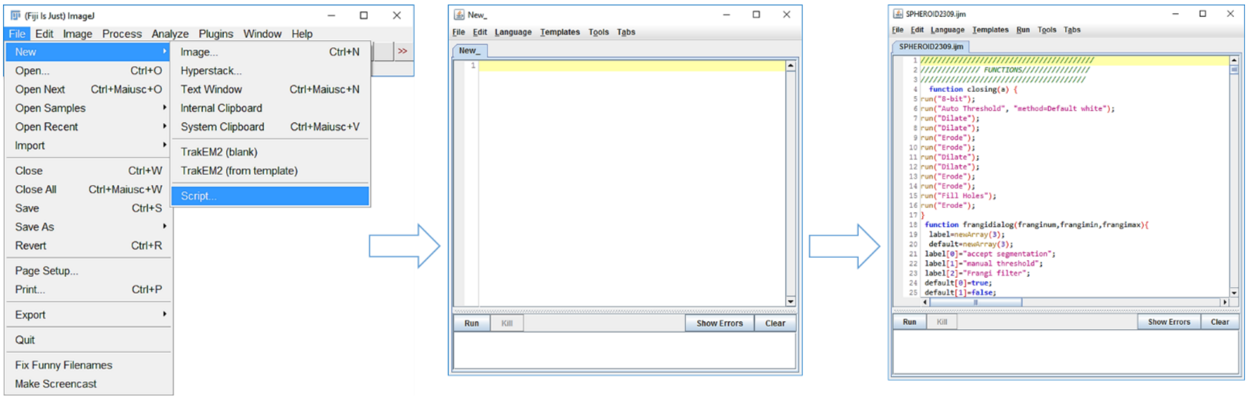

Figure 1 Running the INSIDIA2.0 macro in Fiji.

For a quick guide of INSIDIA 2.0, see Chapter 2. Outputs of INSIDIA are described in Chapter 3. In Chapter 4, details about INSIDIA 2.0 code structure are available.

1.1 INSIDIA 2.0 workflow

INSIDIA 2.0

PART 1: SPHEROID SEGMENTATION (SpS)

Defines spheroid and background (using a threshold), and creates binarized images in a folder

Thresholding can be automatic, manual or based on Frangi filtering

OUTPUT

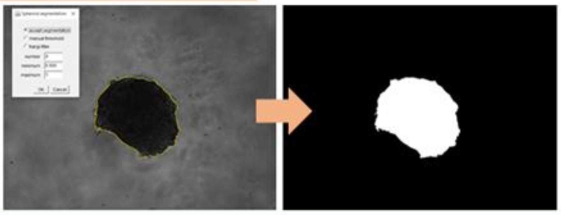

You can skip spheroid segmentation and use other algorithms to binarize your spheroids. Make sure to prepare your folders according to the INSIDIA Guide

PART 2: ANALYSIS

2.1 Parameters from binarized images

- 2.1.1 Shape parameters **UPDATED**
- 2.1.2 Boundary parameters **NEW**
- 2.1.3 Skeletonize parameters **NEW**

2.2 Image Background Normalization & Grayscale images parameters

- 2.2.1 Parameters from image texture (GLCM) **NEW**
- 2.2.2 Parameters from density profiles *Details in [1]*
- 2.2.3 Parameters from density maps *Details in [1]*

PART 3: OUTPUT FILES

- log.txt → *details of input used for the analysis*
- Binarized_parameters.csv → Parameters from *2.1.1, 2.1.2, 2.1.3*
- «file name»_boundary.csv → Boundary coordinates
- Grayscale_parameters.csv → Parameters from *2.2*
- «file name»_profiles.txt → *2.2.2 Details in [1]*
- «file name»_profiles.tif → *2.2.2 Details in [1]*
- Density.tif → *2.2.3 Details in [1]*
- Montage.tif → *2.2.3 Details in [1]*

[1] https://doi.org/10.1002/biot.201700140

Figure 2 INSIDIA workflow

The INSIDIA 2.0 workflow (Fig.2) can be divided into 3 main parts:

- Initially, if necessary, INSIDIA performs **Spheroid segmentation (SpS)** on grayscale images of spheroids. The aims of the SpS are (i) the identification of the spheroid in each image and (ii) the separation from the background by thresholding. The outputs of SpS are binary images containing the spheroid (white pixels) on a black background. Binarized images are saved in a "aaaresults" folder, created during INSIDIA execution, in each experimental folder. Alternatively, the user can provide, together with original grayscale images, binarized (i.e. thresholded) images and skip the execution of SpS part.
- Subsequently, the binarized images are used as input to obtain parameters from binarized images, and specifically
    - Shape parameters, *UPDATED in INSIDIA 2.0*
    - Boundary parameters **NEW* in INSIDIA 2.0*
    - Skeletonize Parameters **NEW* in INSIDIA 2.0*

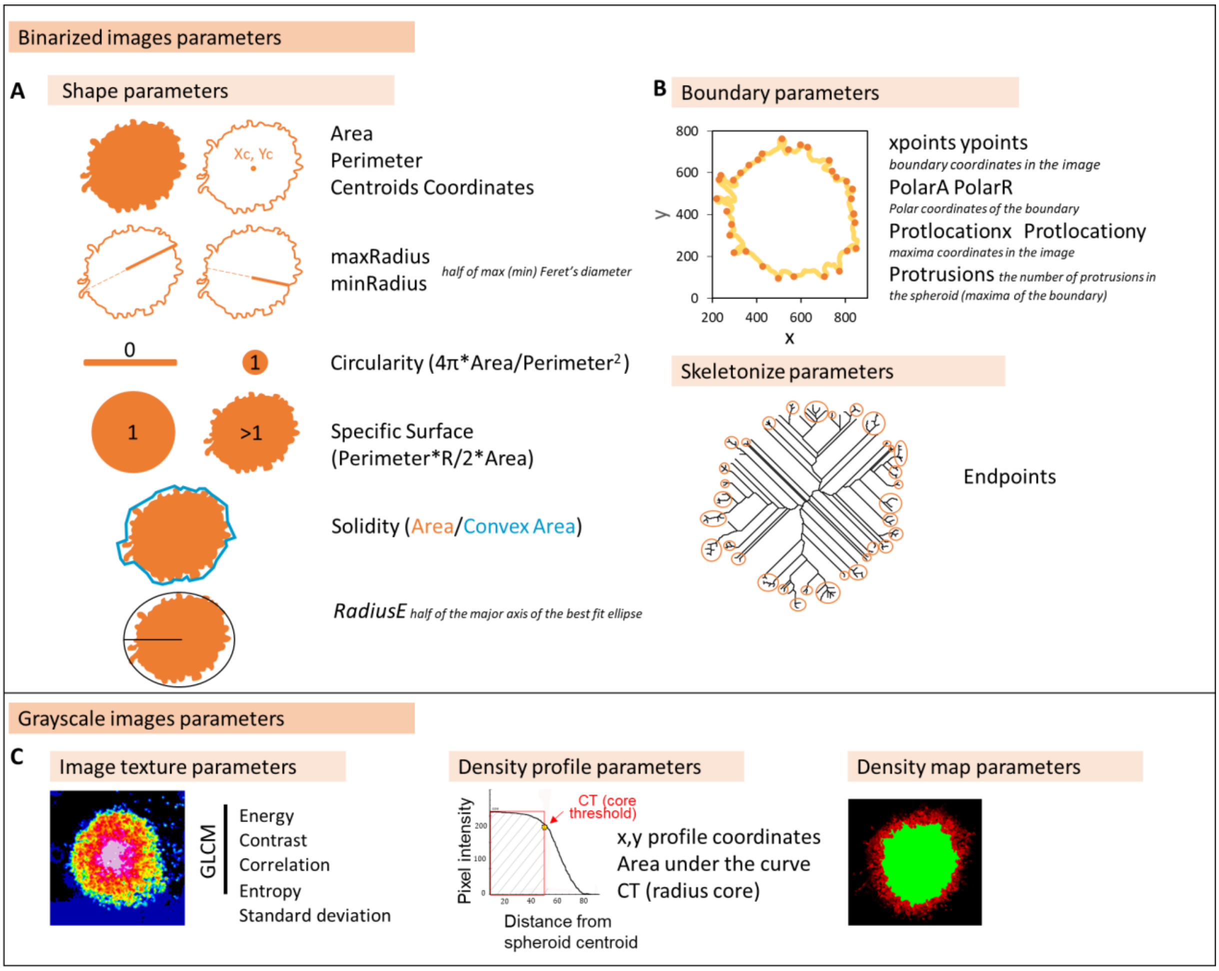

Figure 3 INSIDIA 2.0 Parameters

- After analysis of binarized spheroid, INSIDIA 2.0 proceeds with the analysis of normalized grayscale images and retrieves parameters from image texture. Finally, concentric circles are created from spheroid centroid towards edges to perform **Density Profile** analysis and define the **Core Threshold**, which distinguishes between the spheroid core and edges. Once the CT is calculated, the image is colored accordingly and the **Density Map** of the spheroid is created.

Parameters obtained from INSIDIA are described in detail in Table 1, 2 and 3 in Chapter 3.

1.2 Input images and folders organization

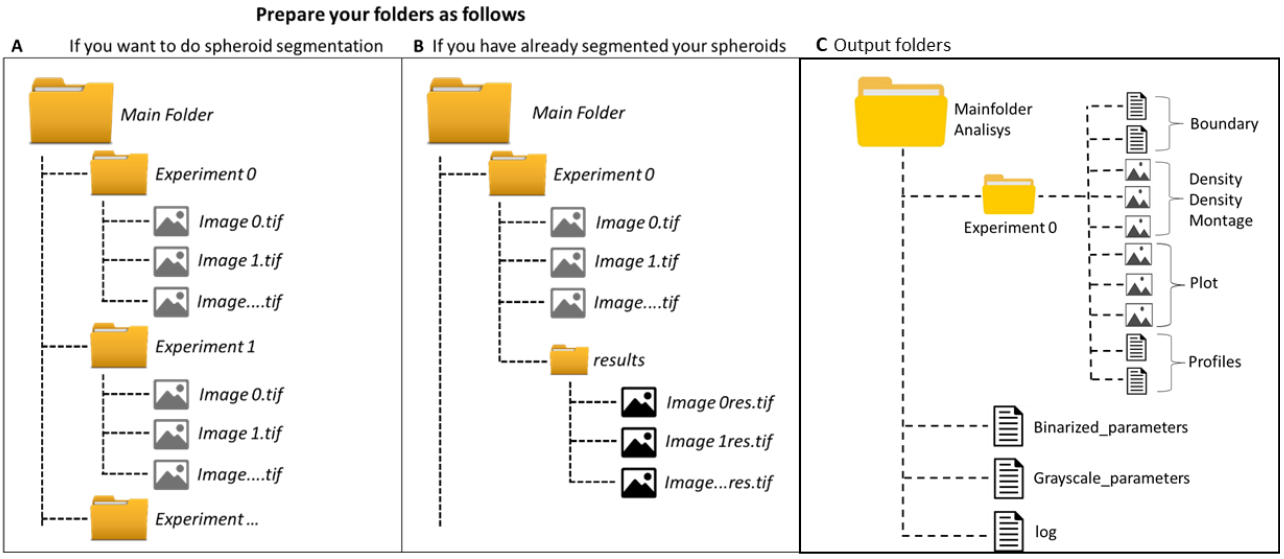

Figure 4 Folders organization (A) Unsegmented spheroids, (B) If user provides segmented images. (C) Macro output

To correctly run INSIDIA, user should provide images in tiff format. The minimal requirement is a "mainfolder" containing a subfolder with images. If you prefer to store your images in several folders, for example, if you want to separate different cell lines, you can add as many subfolders as you want. All the subfolders in the mainfolder will be automatically processed (See Figure 4A).

!Warning! Images and results will be analyzed in alphabetical order.

!Warning! Make sure that images have the same width and height in pixel and are in 8-bit gray.

!Warning! A spheroid in the centre of the image is optimal for the analysis

If the user wants to provide binary images of spheroids and skip the SpS part of the macro, these binary images should be saved in a folder inside each experiment folder (Fig. 4B). Image name should be the same of the corresponding grayscale image with the suffix "res" (Fig. 4B). The binary images should be in 8-bit tiff format and user should verify that the pixel intensity value is 255 for white pixels and 0 for black pixels.

Download mainfolder1 and mainfolder2 from the website for an example of folders organization to perform or skip SpS, respectively.

2. INSIDIA 2.0 QUICK guide

After having organized images as explained in Section 1.2, follow these steps for a quick use of INSIDIA 2.0.

1. Open the macro code as explained in Chapter 1 and click run

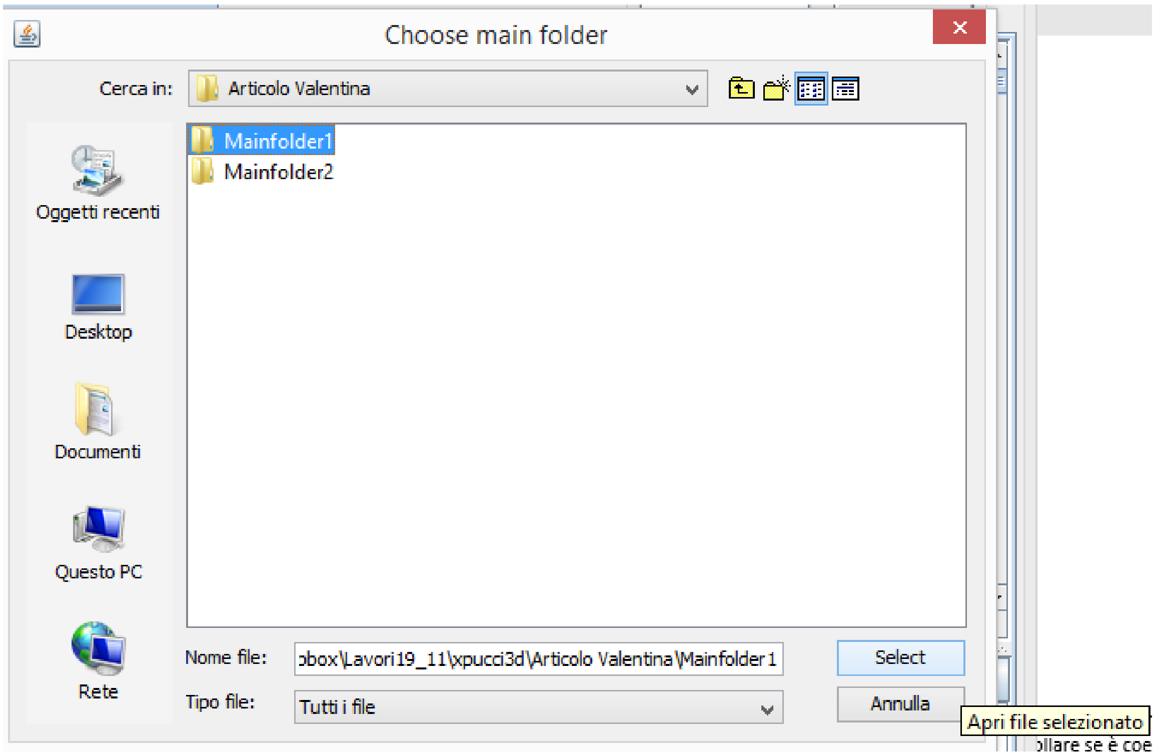

Figure 5 MENU1

2. Go to the Mainfolder location in your computer and click to select it (Fig.5)

!Warning! If the main folder is not organized properly a submenu appears and the macro asks to correct errors in folder organization.

!Warning! Click once, do not open the folder

3. Select one option in the MENU 2 as shown in Fig. 6

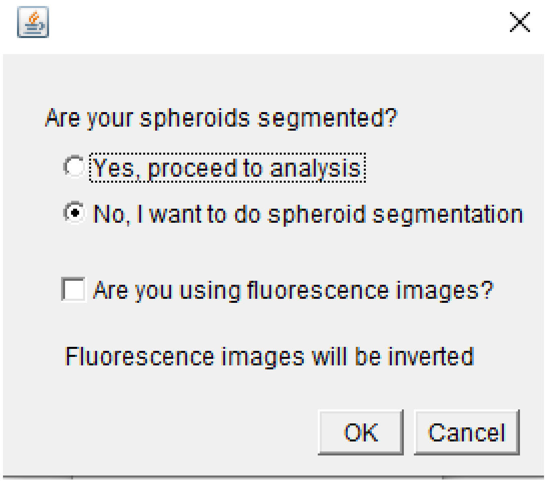

Figure 6 MENU 2

!Warning! Fluorescence images will be inverted

The MENU 2 asks if spheroids are already segmented; if so, select the option *"Yes, proceed to analysis* (hereafter **option 1**). In this case the SpS is skipped and the code proceeds directly to the analysis.

If the spheroid segmentation has not been executed yet, select *"No, I want to do spheroid segmentation"* option (hereafter **option 2**).

4. If you selected **option 1,** the SpS part of INSIDIA is skipped (go to point 5). If you selected **option 2**, the first grayscale image of the first subfolder will be opened and an attempt to automatically retrieve spheroid mass will be done.

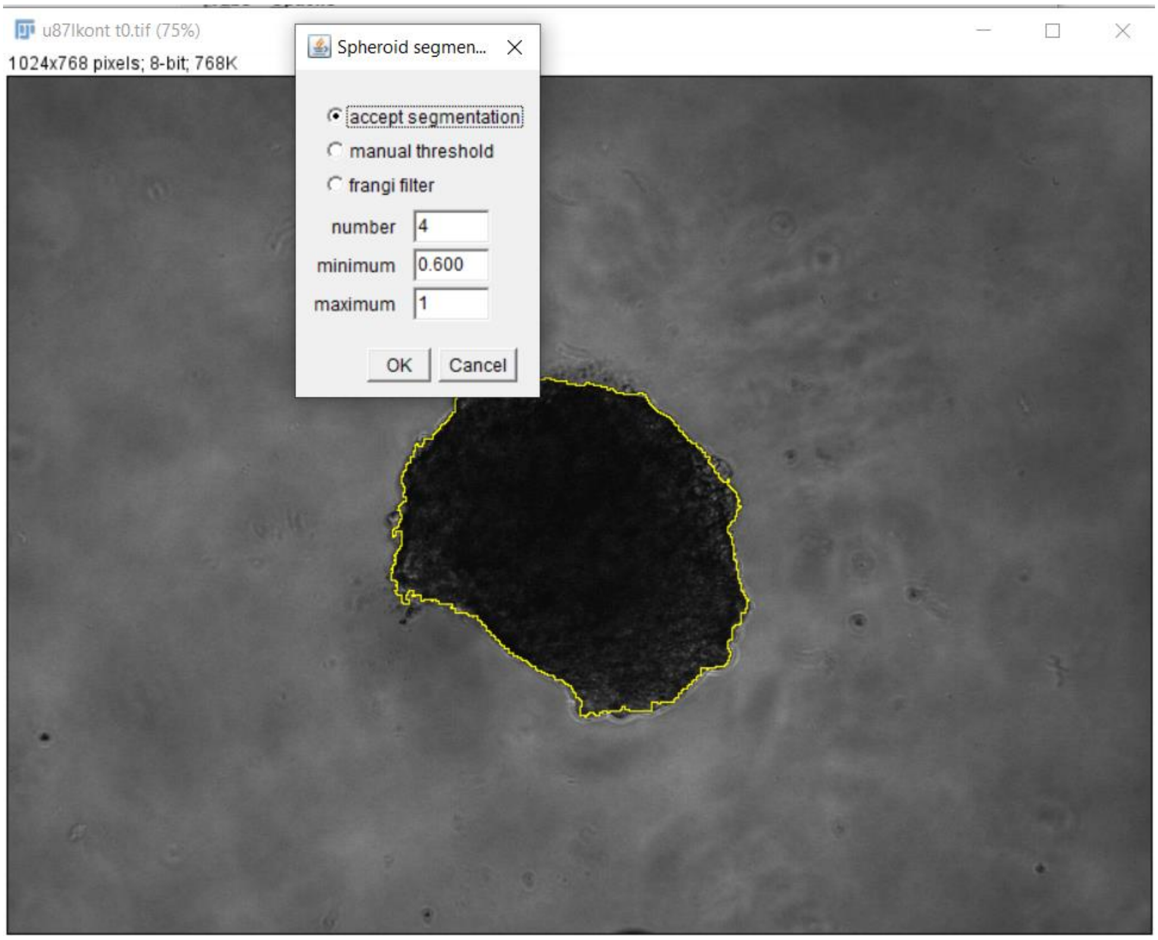

Figure 7 Spheroid Segmentation on a representative grayscale image and Menu SpS.

INSIDIA will display the original grayscale image with the spheroid mass outlined in yellow and a MENU entitled Spheroid Segmentation (Fig.7). In this menu, you can accept automatic thresholding and proceed to the next image. If you are not satisfied with this automatic step, you can perform manual thresholding or Frangi filtering.

If you select manual thresholding a MENU entitled ManualThreshold will appear together with the *Threshold Menu* of ImageJ (Fig.8). In this case, the grayscale image will become blue in some areas. Those areas will be considered as spheroid mass. You can define manually blue regions by using the slide bars in the *Threshold Menu* and change the cut-offs of the histogram. Once satisfied with the blue area considered, click OK in the ManualThreshold menu. INSIDIA will calculate again the spheroid outline (yellow line in Figure 7) and you can accept or repeat this operation.

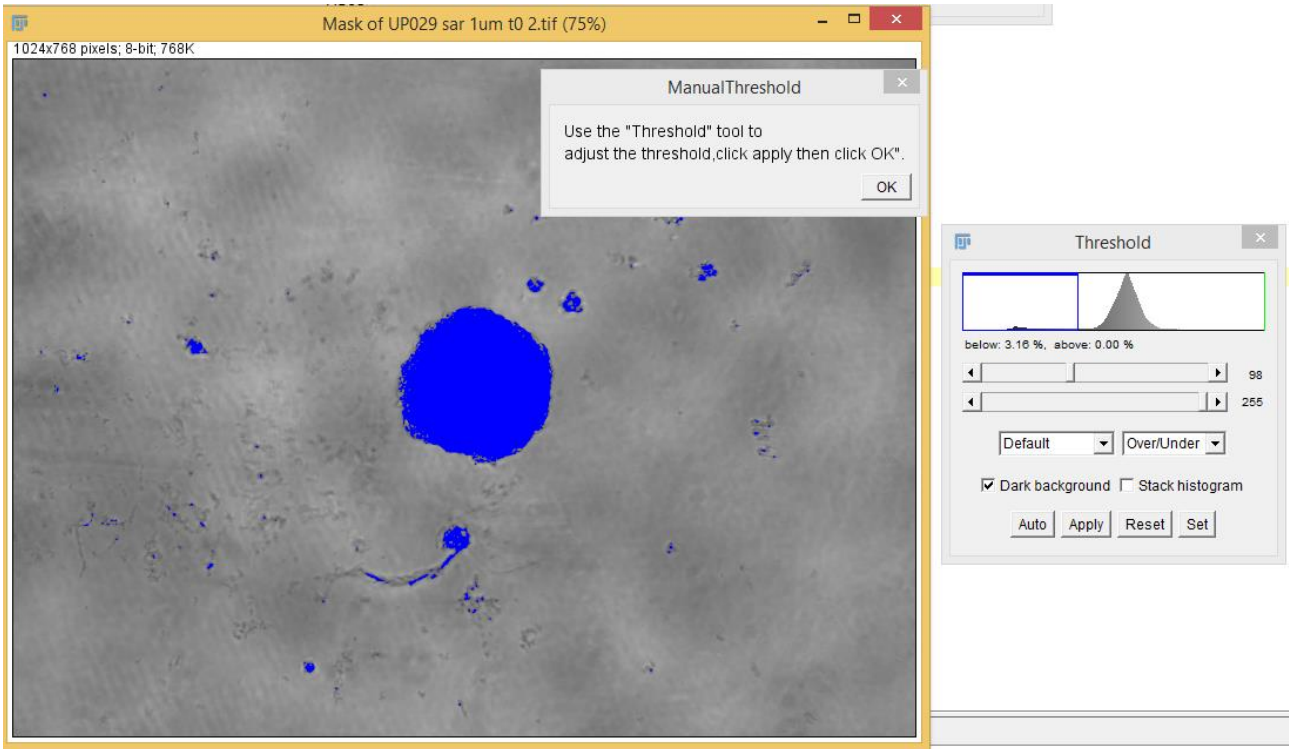

Figure 8 ManualThreshold Menu of INSIDIA with the Threshold menu of ImageJ appear during manual spheroid segmentation

- If you select the Frangi filter, you should provide parameters of number, maximum and minimum in the SpS Menu in Figure7 (see http://imagej.net/Frangi for the meaning of each parameter). INSIDIA will calculate again the spheroid outline and you can accept or repeat this operation.

> !Warning! Frangi filtering might be slow. Wait for completion without clicking on other menus.

The spheroid segmentation is done for all the grayscale images in the subfolders. The binarized spheroids images are saved in a results folder created by the macro. The binarized images will have the same name as the original images with the suffix “res” and will be placed in a results folder in each subdirectory.

> !Warning! In this part, each image is opened, and scale is removed so that units of the image become pixels. This modification is saved on provided

images so if you don't want to lose your data, make sure to use a copy of your image or to save this elsewhere before running the code.

5. The pixel conversion menu (Fig.9) asks if conversion in microns should be applied to output values. If the convert option is unticked the parameters calculated will remain in pixel units.

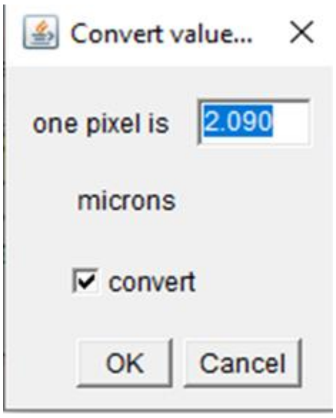

Figure 9 Pixel conversion menu

6. The GLCM texture menu (Figure 10), allows the user to select the step size and the direction of the step for the GLCM analysis [1].

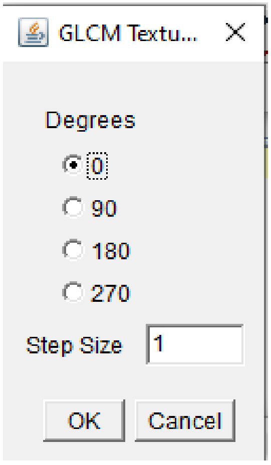

Figure 10 GLCM Texture menu

7. At this point, the macro will calculate all parameters automatically. When the calculation is completed, a final menu will appear and the user clicks OK to terminate the code execution (Fig. 11). The files *created are saved in an analysis folder having name "analysis + the day and time" of the last INSIDIA execution.*

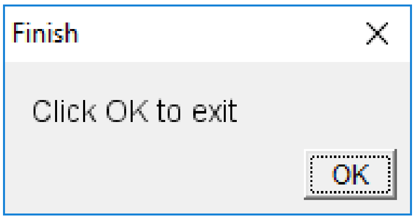

Figure 11 Final menu

> !Warning! Depending on the number of subfolders and images, analysis might take some time. Wait for the final menu without clicking on other menus.

3. INSIDIA output and parameters

After code execution, the analysis folder will contain, a series of folders having the same name as the original subfolder. If we consider the example in Figure 4C, the analysis folder will contain Experiment0, Esperiment1 and so on subfolders. In each subfolder, the density maps, montage, the profiles of the spheroids, the profile plots and the boundary files will be saved. The montage is a visual outcome of the results obtained with the original images (time 0 and last time point) compared to the corresponding density maps. In the main analysis folder, the binarized_parameters, the grayscale_parameters and the log file will be saved. You can open this file in Excel to generate tables and make further calculations and mathematical simulations. In the log file, the pixel to micron conversion adopted, the GLCM texture analysis stepsize and degrees and the tolerance for maxima calculation in the boundary used, are reported.

For the description of parameters see Table 1 (Parameters derived after SpS on Binarized images), Table 2 (Parameters derived from grayscale images) and Table 3 (Parameters derived from Density Map and Density Profiles).

Table 1 Parameters derived from segmented images

	Parameter	*Description*	*Output file*
Shape parameters	Areatotal	Area of the Spheroid (pixel^2)	Binarized_parameters.csv
	Perimeter	Perimeter of the Spheroid (pixel)	

	Xc, Yc	Centre of Mass Coordinates (pixel)	
	maxRadius	Maximum Radius of the Spheroid, calculated as half of max Feret's diameter which is the longest distance between any two points along the selection boundary, also known as maximum caliper (pixel)	
	minRadius	Minimum Radius of the spheroid, is calculated as half of the min Feret's diameter (minimum caliper) (pixel)	
	Circularity	The circularity is calculated with the formula 4π*Area/Perimeter^2 with a value of 1.0 indicating a perfect circle. As the value approaches 0.0, it indicates an increasingly elongated shape.	
	RadiusE	Radius calculated using the best fit ellipse (half of the major axis) (pixel)	
	SpecSurf	Specific surface: the ratio of the perimeter over its total area, is employed to quantify the degree of "fingering" of the growing tumour. For a perfectly circular shape with radius R, the associated specific surface is given by 2/R, which is the minimum value among all shapes with the same area. (pixel)	
	Solidity	Area/Convex area, the solidity measures the density of an object	
Boundary parameters	Protrusions	the number of protrusions in the spheroid TOLERANCE	Binarized_parameters.csv
	xpoints ypoints	boundary coordinates in the image(pixel)	Boundary.csv
	PolarA PolarR	Polar coordinates of the boundary (radiants,pixel)	
	Protlocationx Protlocationy	maxima coordinates in the image (pixel)	
Skeletonize parameters	endpoints	endpoints of the skeletonized image of the spheroids	Binarized_parameters.csv

Table 2 Parameters derived from image grayscale texture

Parameter	*description*	*output*
Energy	Energy is a measure of image homogeneity. High energy value is present when the image is made of pixels with very similar gray level values, which points to the texture that is uniform and	

	contains repetitive structures.	Grayscale parameters
Contrast	Contrast is a measure of image heterogeneity, and represents the measure of gray level variation and spatial disorder. High CON values can be found in heavy textures images.	
Correlation	Correlation is a measure of gray level linear dependence between the pixels at the specified positions relative to each other.	
Entropy	Entropy is a measure of image heterogeneity, and represents the measure of gray level variation and spatial disorder. High ENT indicates that the image texture is statistically more chaotic.	
Stdev	Standard deviation of the gray values.	

Table 3 Parameters derived from Density Profile and Density Map

Variable	*Parameter Description*	*output*
X,Y	Coordinates of the profiles, conversion is applied to the X coordinates. Y coordinates represent normalized intensities<br>(X pixel, Y intensity)	Profiles
ACMtotal	Area under the density profile curve	
ACMcore	Area under the density profile curve below the CT	
ACMinvasion	Area under the density profile curve over the CT	
ACMPercCore	(ACMcore/ACMtotal)*100	
ACMPercInvasion	(ACMinvasion/ACMtotal)*100	
RadiusCore	Core Radius obtained with CT(pixel)	
AreaCore	Area of pixel with intensity>CT(pixel^2)	
AreaInvasion	Area of pixel with intensity<CT(pixel^2)	
PercCore	AreaCore /AreaTotal*100	
PercInvasion	AreaInvasion /AreaTotal*100	
PeriMap	Perimeter of DensityMap Core	

!Warning! In Table 1,2,3, Standard units of measurement are reported for each parameter (pixel/pixel^2), if the user checks the box **convert** in the menu in Fig.9 the units of measurement will be converted.

4. INSIDIA Code

In this chapter, the code is described to allow the user to understand and personalize INSIDIA 2.0.

4.1 Functions (lines 1-39)

In the first lines of the code, functions are defined. Do not modify this part.

1) The function *closing* performs the morphological operation of closing, which is a series of dilations and erosions of binary images to define the contour of an object (in this case the spheroid) (see https://en.wikipedia.org/wiki/Closing_(morphology) for further details). During this operation, black holes inside the object are filled in white.

2) The function *error* creates error messages.

3) The function *largest* is a function that runs *Analyse particles* on binary images and leaves only the largest particle in the image. It is used in INSIDIA to segment the spheroid and eliminate noise.

4.2 ImageJ settings & Errors (lines 40-51)

In this section, images previously opened are closed; Roi Manager is reset, the type of Measurements are set and the background colour is set to black. Also, the error messages are created, and the date and time of code execution are stored in variables.

4.3 Menu & Folders (lines 52-101)

This part of the code is necessary to create the first menu of the macro and calculate the number of folders and images.

4.4 INSIDIA PART 1: Spheroid Segmentation (lines 102-239)

This part of the code performs Spheroid Segmentation on all the images in the subfolders and is executed only if, in the Menu 1, option 2 has been selected.

In this part, each image is opened, and scale is removed so that units become pixels. The image is transformed into 8-bit image and the height and width of the image are stored in the variables h and w, respectively.

Then the *"automatic thresholding"* with ImageJ default method is performed. Details on this operation can be found at http://imagej.net/Auto_Threshold. This threshold is performed since in most cases it can easily recognize the spheroid in the image. The automatic thresholding creates a binarized image with recognized objects in white and the background in black.

After this operation, the *"Analyze particles"* plugin is used to select the largest white object in the binary image, the selection is stored in the *"ROI Manager"* and is used to define a yellow outline on the greyscale image.

INSIDIA will display the original grayscale image with the spheroid mass outlined in yellow and Menu entitled Spheroid Segmentation. In this menu, you can accept automatic threshold and go to the next image. If you are not satisfied by the spheroid segmentation you can perform manual thresholding or Frangi filtering as explained in Chapter 2. In manual thresholding, the Threshold tool of ImageJ is opened, and the macro waits for a user manual set of the threshold. In the Frangi filtering option, this filter is applied to the image considering the parameters specified in Menu SpS.

Once that SpS is accepted, the image is saved in the results folder as "tiff" and INSIDIA proceeds to the following image/folder. INSIDIA will display again original grayscale image with the spheroid mass outlined in yellow and Menu SpS.

4.6 INSIDIA PART 2: Analysis

4.6.1 Parameters (240-278)

Empty arrays for storing variables are created.

4.6.2 GLCM Menu (279-286)

Creation of Menu for GLCM texture analysis.

4.6.3 Shape Parameters from binarized images (287-363)

Thresholded images in the results folder are opened and the shape parameters (Table 1) are retrieved.

4.6.4 Boundary Parameters from binarized images (364-413)

Boundary of binarized spheroid is analysed and the boundary parameters (Table 1) are retrieved. Parameters obtained are saved in a separate file having suffix Boundary.csv for each image

4.6.6 Skeletonize Parameters from binarized images (414-422)

Binarized spheroid is skeletonized and skeletonize parameters (Table 1) are retrieved.

4.6.7 Image Normalization and background removal (423-453)

!Warning! The CT and all the following operations are performed on inverted images, i.e. the darkest pixel (0) becomes the lightest (255). In this situation, the spheroid becomes the lighter element in the image, see the example in the Fig.12 During this operation, the intensity values of the image are not modified.

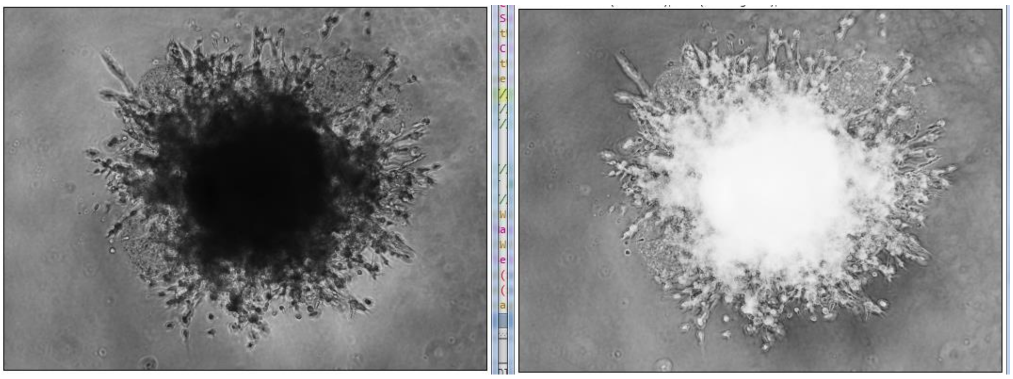

Figure 12 Inverted LUT on a representative spheroid image

During Image Normalisation, to correct for microscope illumination, the background of the image is subtracted. This is done by averaging the grey values outside the spheroid mass. The area outside the spheroid mass is obtained by selecting the black pixels in the SpS image (binarized) and creating a mask. Note that this mask will be applied also to grayscale images to calculate density profile. The core thresholding is necessary to determine the intensity value (CT) that determines which part of the spheroid should be considered core (pixel intensity> CT) or invasive edge (pixel intensity< CT).

4.6.7 GLCM texture analysis (454-462)

Here the GLCM plugin is used and parameters in Table 3 are retrieved.

4.6.8 Saving of normalized images (463-476)

4.6.9 Pixel Conversion Menu (476-491)

This part of the code is necessary to create the Pixel Conversion menu. If convert is not checked conversion doesn't occur and the pixel conversion value

4.6.10 Density Profile (492-540 and 561-607)

The Density profile plot is created by using the Concentric circles plugin with a circle line width of 1 pixel and starting from a circle of 1 pixel radius and going to the maximum radius equal to the maxRadius parameter.

The density profile plot is used to define the parameters of the totalArea under curve (ACMtotal) which is further divided in ACMcore and ACMedges that are respectively, the area under the curve portion below the CT value (the core) and over the CT value (the edges). These areas are calculated by mathematical integration. In the profile.txt file, the y profiles are printed after each file name and the x values are reported at the end.

4.6.11 CT Calculation (541-560)

This process defines a limit between core and invasive edges automatically from the density profile. The profiles start with a plateau and then display a decreasing slope. The plateau corresponds to the quite uniform value of pixel's intensity in the spheroid's core. As moving away from the centroid, the Intensity starts decreasing. The limit of the core (CT) is the last intensity value of the curve plateau.

To calculate CT, the script iteratively draws a rectangle around the profile defined by a line parallel to x axis drawn at the maximum intensity (average of the first 10 points of the profile) and a moving vertical line parallel to y axis. The vertical line first crosses the last point of the profile and then is traced, at each loop, 5 points closer to the centroid (located at axis origin). Each time the integral of the curve between axis origin and y line is calculated

and is compared to the bounding rectangle area. When the y line is traced at the CT, the difference between the area under the curve is approaching to the area of the rectangle (in the code > 95%). At this point the CT is chosen. This value leads to the distinction of the core (the area under the curve below CT) from the invading edges (the area under the curve above CT). For more details see the publication [2].

4.6.12 Density Map & Montage (608-704)

In these code lines, the Density Map is created. Briefly, each intensity value of the image is read compared to the CT: if the intensity is >CT the pixel is set to green and will be part of the core. The sum of green pixels will be the CoreArea. Conversely, values with intensity<CT will be in red and will be the invasive edges. The area of invading edge is calculated as the difference between the TotalArea and the CoreArea. The maps are saved in the corresponding subfolders.

4.7 INSIDIA PART 3: Output

Output, Save & exit (705-752)

In this section, the output files are produced, and the values are converted in microns if required by the user. Folders are organized and a final confirm Menu is created.

References

[1] A. Hladnik, G. Krumpak, M. Debeljak, D.G. Svetec, Assessment of paper surface topography and print mottling by texture analysis, in: Proc. ImageJ User Dev. Conf. Luxemb., 2010.

[2] C. Moriconi, V. Palmieri, R. Di Santo, G. Tornillo, M. Papi, G. Pilkington, M. De Spirito, M. Gumbleton, INSIDIA: A FIJI Macro Delivering High-Throughput and High-Content Spheroid Invasion Analysis, Biotechnol. J. 12 (2017). doi:10.1002/biot.201700140.
