# Supplementary material for: INSIDIA 2.0 High-Throughput Analysis of 3D Cancer Models: Multiparametric Quantification of Graphene Quantum Dots Photothermal Therapy for Glioblastoma and Pancreatic Cancer
Source: Int J Mol Sci. 2022 Mar 16;23(6):3217. doi: 10.3390/ijms23063217 (PMC8948775; doi:10.3390/ijms23063217)
Supplement: Supplementary file 1 [file ijms-23-03217-s001.zip › Supplementaary Figures.pdf]

Supplementary Material

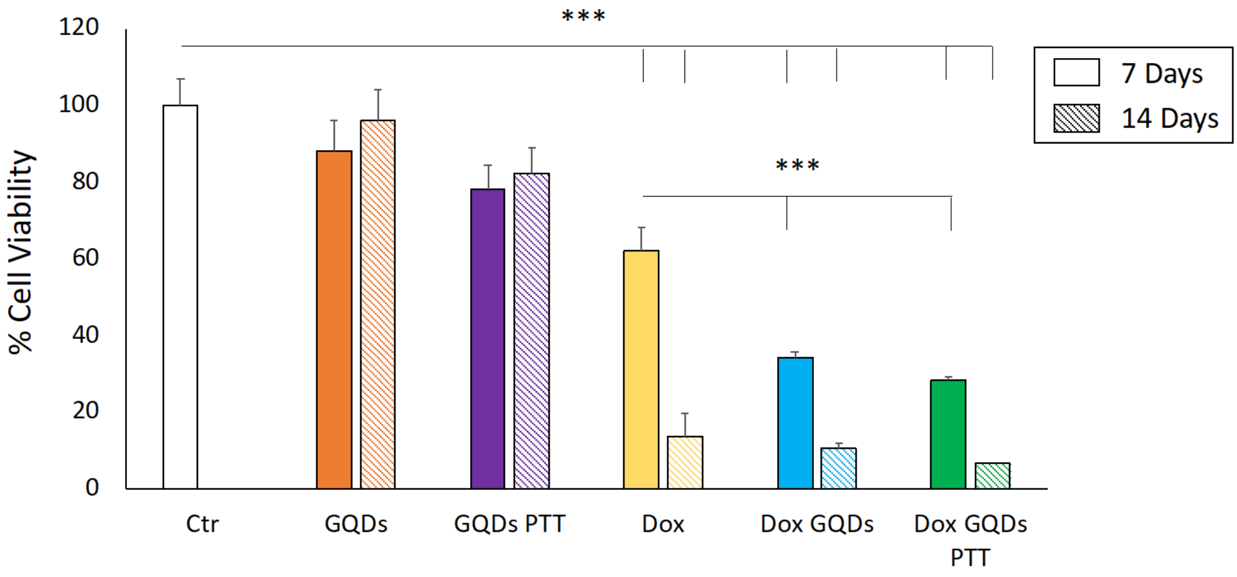

Figure S1. Viability of U87 Spheroids after 7 or 14 days of treatment normalized to control untreated samples (Ctr). Asterisks indicate p<0,05.

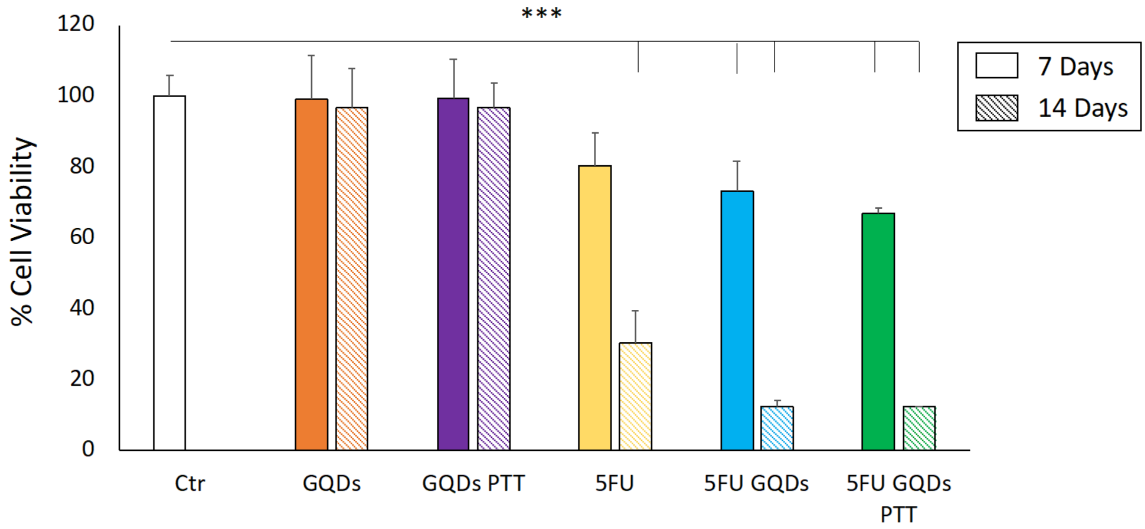

Figure S2. Viability of PANC-1 Spheroids after 7 or 14 days of treatment normalized to control untreated samples (Ctr). Asterisks indicate p<0,05.

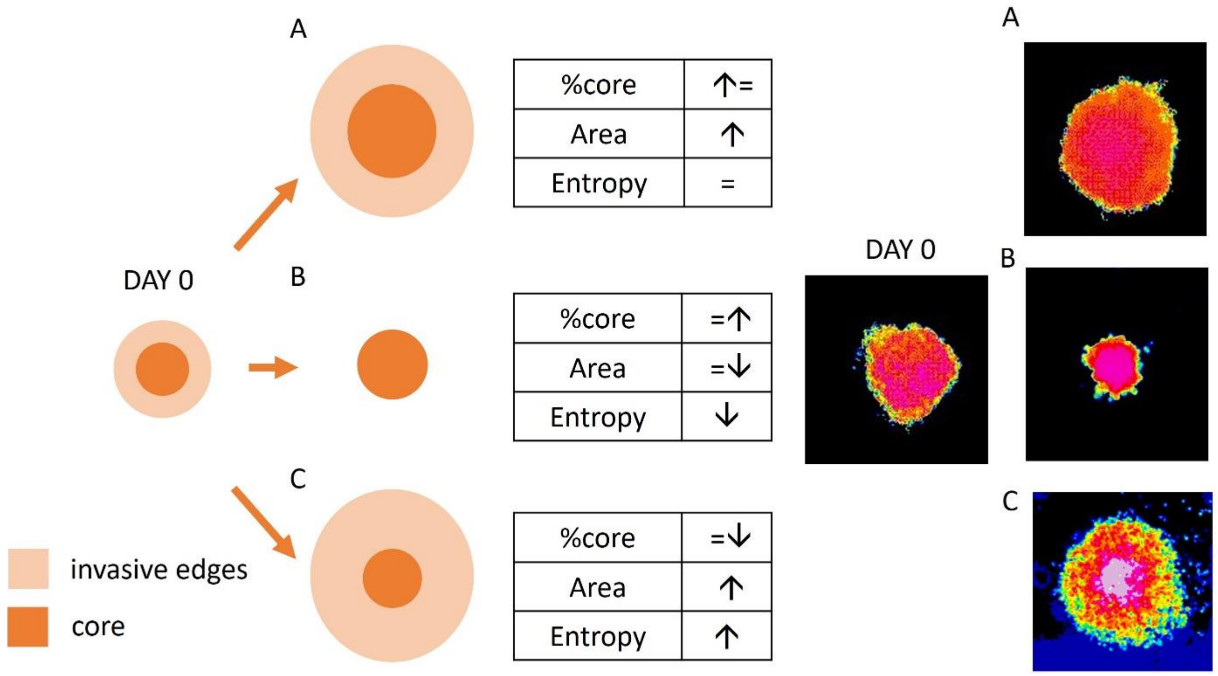

Figure S3. Characteristic parameters used for analysis of spheroids disruption are core percentage, Area and Entropy. In this figure, we show the trend of these parameters in spheroid growth (A), collapsing spheroid with loss of invasive edges (B), and spheroids disrupted with loss of integrity (C).
